# Mr. Jowett's Case of Bronchitis Succeeded by Pneumonia and Pericarditis

**Published:** 1826-07

**Authors:** 


### CASE OF BRONCHITIS FOLLOWED BY PNEUMONIA AND PERICARDITIS.*

October, 15th, 1825, Bryan Flinn, ætat. 35, labours under acute bronchitis.

*Ausculltic indications. Sonorous* and *sibilant rattles,* generally audible throughout the thorax; in some spots verging to the *mucous*

* Mr. Jowett, New Ed. Journal.

kind. *Respiration prolonged* and blowing, at times inaudible in spaces of various extent: it reappears in general after a fit of coughing. *Percussion.* Sound *good.* He expectorates in the day about a pint of clear, viscid, and rather frothy mucus, and is too weak to bear sanguineous depletion; legs and feet œdematous. Blisters to the sternum, and ¼ of a grain of tartarized antimony every four hours. Spare diet and rest.

Under this treatment he was gradually recovering, but November 16th, he was seized with dull pain in the left side of the chest, and other symptoms of pneumonia; legs again œdematous. 22d. Pain gone: the dyspnœa and cough oblige him to sit up constantly: sputa opake and gelatinous, the froth thick and in quantity. *Percussion.* The sound good in the right back, but dead in the left. *Stethoscopy.* No respiration audible at the bottom of the left side of the chest posteriorly, but only a weak rattle on inspiring forcibly. More or less bronchial respiration, with resonance of voice nearly resembling egophony, between the left scapula and the spine, around and over the inferior half of the scapula, and along the fifth rib towards the sternum. Respiration vesicular, but feeble, posteriorly, at the summit of the lung, anteriorly, down to the third rib; below this inaudable. Tartarized antimony to be continued. These symptoms were relieved, and he appeared to improve till November 26th, when he was seized with a great sense of constriction about the heart, forcing him to sit up constantly. *Stethoscopy.* The heart's action natural as to rhythme, but it appeared to be *at a distance* from the side. Leeches and a blister to the præcordial region. 28th, Better. 29th, He can lie lower in bed, but still that intolerable sensation about the heart: pulse weak and rather irregular. Besides the former indications by the stethoscope, a *bubbling noise* was distinctly heard at intervals; it seemed to accompany and be caused by the motions of the heart. At night it was so distinct as to be heard by a patient in the next bed. The next morning it was gone. He was free from pain from this time till December 3d, when the feelings about the heart returned. *Stethoscopy.* The heart was felt beating in a confused manner in a considerable præcordial space. Pulmonary indications nearly the same. December, 10th, Œdema appeared again, and advanced till the 20th, when he died.

*Dissection,* 28 hours after death. Present, Dr. Marshall Hall, Dr. Godfrey Howitt, Mr. Oldknow, Mr. Cursham, to whom, prior to opening the body, Mr. Jowett read the following note.

"21st December.—On looking over my notes of Flinn's case, for the purpose of forming an opinion of the morbid appearances, before examining the body, it appears, that the inferior lobe of the left lung is generally in a state of hepatization (probably of the grey kind): whether there be (any) pleuritic effusion, and to what extent I cannot say; I think it cannot be abundant. Although not quite certain, yet in this case, I do expect to find pericarditis, of course accompanied with effusion into the pericardium."

On opening the thorax, the pericardium was found very large, extending as high as the second left rib, and more than usual laterally. It contained some air, and two pints and a half of thin opake greenish yellow pus, mixed with flakes of coagulable lymph, by a thick recent layer of which the internal surface of the pericardium and external surface of the heart were coated. The heart itself of moderate size, its parietes thin, pale and flabby. It adhered posteriorly to the pericardium, and was pushed upwards and backwards.

The lungs were firmly united to the walls of the thorax by old adhesions, which were, for the most part, infiltrated with transparent, and nearly gelatinous fluid—no pleuritic effusion. The superior lobe of the left lung not so crepitous as natural; when incised, much serous fluid, with air, oozed out. This was the case with the right lung. The inferior lobe of the left lung was dense, fleshy, and of tender consistence: it was in a state intermediate between the red and grey hepatization, more approaching the latter. The lower part of the trachea, and the bronchial tubes contained much thick, frothy, mucus. The mucous membrane reddened throughout both lungs, not so in the inferior lobe of the left one.

*Abdomen.* Some old adhesions between the omentum and peritoneum; also between the liver, which was large and gorged with blood, and the diaphragm. Some serous effusion into the abdominal cavity.

*Head,* not examined.—*Mr. Jowett, Ed. Journ. Med. Science, No. 2.*

We perfectly agree with the able and indefatigable author of the above case, that the utility of auscultation and percussion in the diagnosis of diseases of the lungs, is strongly illustrated by it. Our readers will find a still more remarkable and interesting case by the same author in another part of this Number.

---

POPLITEAL ANEURISM.*

